# Tools for translational epigenetic studies involving formalin-fixed paraffin-embedded human tissue: applying the Infinium HumanMethyation450 Beadchip assay to large population-based studies

**DOI:** 10.1186/s13104-015-1487-z

**Published:** 2015-10-06

**Authors:** Ee Ming Wong, JiHoon E. Joo, Catriona A. McLean, Laura Baglietto, Dallas R. English, Gianluca Severi, John L. Hopper, Roger L. Milne, Liesel M. FitzGerald, Graham G. Giles, Melissa C. Southey

**Affiliations:** Department of Pathology, The University of Melbourne, Melbourne, VIC 3010 Australia; Anatomical Pathology, Alfred Health, The Alfred Hospital, Melbourne, VIC 3181 Australia; Cancer Epidemiology Centre, Cancer Council Victoria, 615 St Kilda Road, Melbourne, VIC 3004 Australia; Centre for Epidemiology and Biostatistics, The University of Melbourne, Melbourne, VIC 3010 Australia

**Keywords:** Population-based translational epigenetic studies, Formalin-fixed paraffin-embedded, DNA methylation, Epigenetics, HM450K beadchip, Breast tumour subtype, *BRCA1*

## Abstract

**Background:**

Large population-based translational epigenetic studies are emerging due to recent technological advances that have made molecular analyses possible. For example, the Infinium HumanMethylation450 Beadchip (HM450K) has enabled studies of genome-wide methylation on a scale not previously possible. However, application of the HM450K to DNA extracted from formalin-fixed paraffin-embedded (FFPE) tumour material has been more challenging than application to high quality DNA extracted from blood. To facilitate the application of this assay consistently across a large number of FFPE tumour-enriched DNA samples we have devised a modification to the HM450K protocol for FFPE that includes an additional quality control (QC) checkpoint.

**Results:**

QC checkpoint 3 was designed to assess the presence of DNA after bisulfite conversion and restoration, just prior to application of the HM450K assay. DNA was extracted from 474 archival FFPE breast tumour material. Five samples did not have a detectable amount of DNA with an additional 42 failing to progress past QC checkpoint 3. Genome-wide methylation was measured for the remaining 428 tumour-enriched DNA. Of these, only 4 samples failed our stringent HM450K data criteria thus representing a 99 % success rate. Using prior knowledge about methylation marks associated with breast cancer we further explored the quality of the data. Twenty probes in the *BRCA1* promoter region showed increased methylation in triple-negative breast cancers compared to Luminal A, Luminal B and HER2-positive breast cancer subtypes. Validation of this observation in published data from The Cancer Genome Atlas (TCGA) Network (obtained from DNA extracted from fresh frozen tumour samples) confirms the quality of the data obtained from the improved protocol.

**Conclusions:**

The modified protocol is suitable for the analysis of FFPE tumour-enriched DNA and can be systematically applied to hundreds of samples. This protocol will have utility in population-based translational epigenetic studies and is applicable to a wide variety of translated studies interested in analysis of methylation and its role in the predisposition to disease and disease progression.

**Electronic supplementary material:**

The online version of this article (doi:10.1186/s13104-015-1487-z) contains supplementary material, which is available to authorized users.

## Background

Recent technological advances have made genome-wide studies of epigenetics possible on a scale suitable for large population-based studies (akin to what SNP chips enabled for genome-wide association studies). For example, the Infinium HumanMethylation450 Beadchip (HM450K) has enabled studies of genome-wide methylation on a scale not previously possible. However, application of the HM450K to DNA extracted from formalin-fixed paraffin-embedded (FFPE) tumour material has been more challenging than application to high quality DNA extracted from blood.

Archival FFPE tumour material represents a precious resource for many large epidemiological studies. However, the use of this material is often challenging as DNA extracted from FFPE tumour material is frequently of low quantity and highly degraded [1], thus making it difficult to obtain consistent molecular analysis across a large number of samples.

Genome-wide detection methodologies based on next-generation sequencing and microarrays applied to FFPE tumour-enriched DNA have the potential to address many of the current significant research questions being pursued in translational epigenetic studies. However, most of these platforms require large amounts of high quality DNA. Alternate protocols are therefore needed to address the issues of DNA quantity and quality associated with DNA extracted from FFPE tumour material so that the potential of combining these platforms and resources can be realised for translational studies.

We have previously shown that DNA extracted from dried blood spots can be successfully applied to the HM450K platform. High quality and reproducible results were obtained from DNA extracted from matched archival dried blood spot and frozen buffy coat (correlation coefficient r > 0.99) [2]. Although more recent studies have demonstrated the reliable application of the HM450K platform to FFPE tumour-enriched DNA compared with DNA extracted from fresh frozen material [3, 4], they do not address the issues faced by researchers wanting to apply this assay to large population studies. Moran and colleagues measured methylation on the HM450K platform in DNA extracted from matched fresh frozen tumour and newly fixed FFPE tumour material. This work addressed the effect of formalin but it did not consider the possible effect that long term storage of FFPE tumour material might have on DNA quality and assay output [3]. This issue was considered by Dumenil and colleagues who compared DNA extracted from fresh frozen material with FFPE tumour-enriched DNA that was stored between 4 and 19 years (storage conditions unspecified). Based on the signal of the 3000 most differential probes between the two DNA types, they found that the difference in methylation signals between FFPE tumour-enriched DNA and DNA extracted from fresh frozen material correlated with the length of storage time [4].

In both studies, FFPE tumour-enriched DNA was restored with the recommended Infinium HD FFPE Restore protocol (Illumina, San Diego, CA, USA) that has been shown to improve the performance of FFPE tumour-enriched DNA on the HM450K [5, 6]. Highly correlated methylation values were observed between DNA extracted from matched fresh frozen and restored FFPE tumour-enriched DNA compared with unrestored FFPE tumour-enriched DNA (r > 0.91 vs r > 0.81). Additionally, highly reproducible data were obtained from restored FFPE tumour-enriched DNA (r = 0.99) compared with their unrestored counterparts (r = 0.90) [5]. The same observations were reported when Siegel and colleagues compared the restoration protocol to an alternate ligation method (using the REPLI-g ligase) in DNA extracted from FFPE tumour material and matched fresh frozen material [6].

All four studies described above used relatively moderate quantities of DNA (100–500 ng) as starting material for bisulfite conversion, followed by the Infinium restoration and HM450K protocols [3–6]. However, obtaining this amount of DNA is challenging to achieve consistently across a large number of archival FFPE tumour samples. Further, these reports did not measure the presence of FFPE tumour-enriched DNA after bisulfite conversion and restoration, making the subsequent performance of these samples on the HM450K platform unpredictable.

**Fig. 1:**
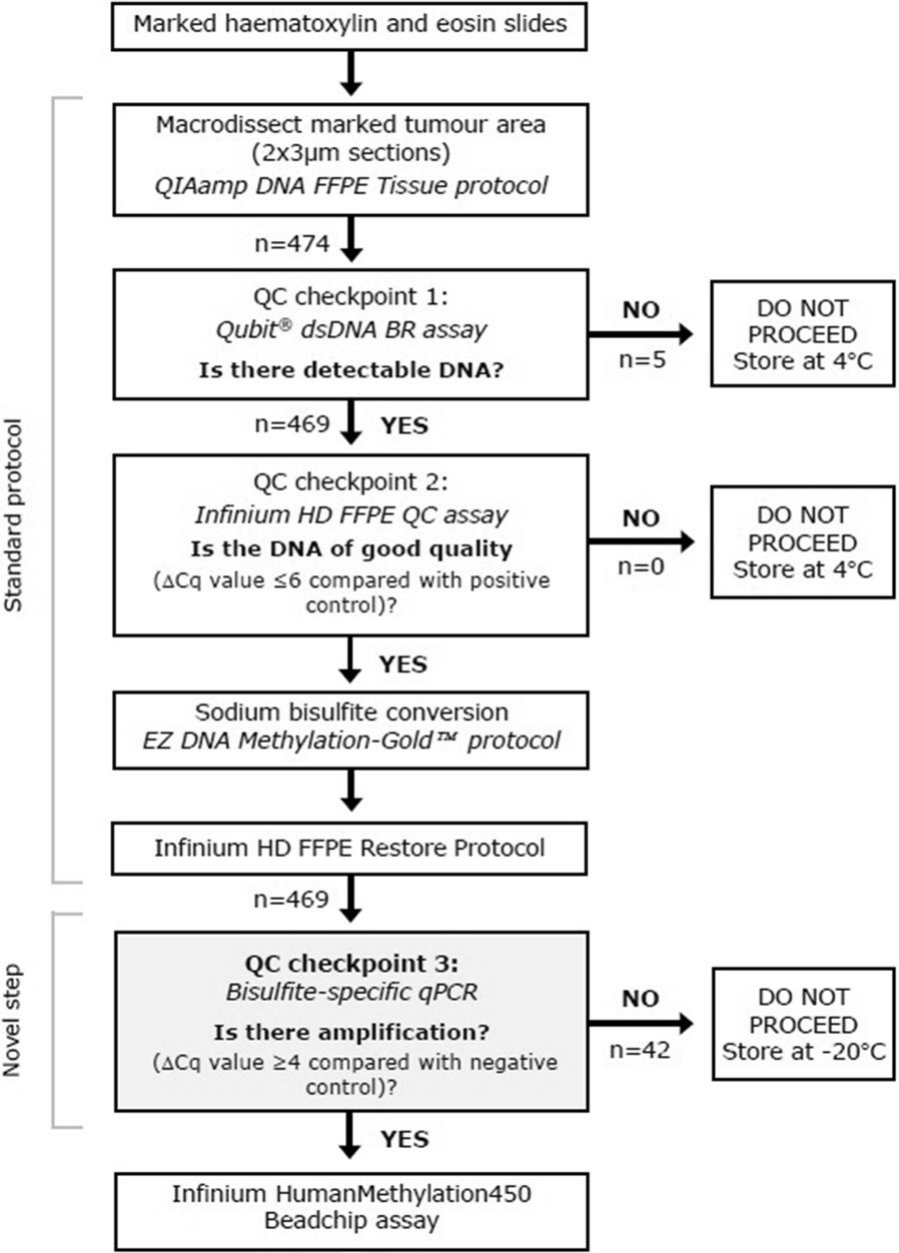
Workflow diagram for assessing the suitability of FFPE tumour-enriched DNA on the HM450K platform. A novel checkpoint (QC checkpoint 3) has been incorporated in addition to the standard protocol to assess DNA availability after sodium bisulfite modification and restoration. The number of samples entering and exiting each QC checkpoint is as indicated

To address these issues, we adopted a modified protocol (Fig. 1) based on the recommended Illumina Infinium workflow for FFPE tumour-enriched DNA which incorporated a third quality control (QC) checkpoint in the form of an in-house designed bisulfite-specific qPCR assay to assess the capacity of the protocol to support large population-based translation epigenetics studies.

## Methods

### Melbourne Collaborative Cohort Study

The study was performed on material from 474 breast cancer affected female participants of the Melbourne Collaborative Cohort Study (MCCS). The MCCS is a prospective study of more than 40,000 men and women aged 40–69 years at baseline who were recruited between the years 1990–1994 [7]. Pathology material related to each breast cancer case had previously been retrieved from the diagnostic service laboratory and reviewed by qualified pathologists. Representative haematoxylin and eosin (H&E) stained sections and unstained sections (3 μm) had been prepared and stored desiccated at 4 °C for up to 15 years. Immunohistochemical staining and breast cancer subtyping were conducted as described by Blows et al. [8]. Written informed consent was obtained from each participant and the study was approved by the Human Research Ethics Committee of Cancer Council Victoria [7].

### DNA extraction from FFPE breast tumour material

The tumour area most suitable for macrodissection was identified by a qualified pathologist (CAM) and recorded by directly marking up the representative H&E stained section. Macrodissection of FFPE breast tumour material was performed on an average of two 3 μm unstained sections (corresponding to the marked H&E stained section). DNA was extracted using the QIAamp DNA FFPE protocol (Qiagen, Hilden, Germany) as per the manufacturer’s instructions except that the tumour material was incubated in Buffer ATL at 56 °C for 48 h, with 20 ul of Proteinase K (20 mg/ml) replenished at 0 and 24 h to increase the digestion of proteins. FFPE tumour-enriched DNA was eluted twice in Buffer ATE to achieve a final elution volume of 20 μl.

### QC checkpoint 1: Qubit^®^ dsDNA BR assay

The presence of FFPE tumour-enriched DNA was measured using the Qubit^®^ dsDNA BR Assay kit on the Qubit^®^ Fluorometer (Life Technologies, Carlsbad, CA, USA) as per manufacturer’s instructions. Samples with undetectable DNA amounts were not further progressed through the workflow.

### QC checkpoint 2: Infinium HD FFPE QC assay

The quality of FFPE tumour-enriched DNA was assessed using the Infinium HD QC assay (Illumina, San Diego, CA, USA) on the LightCycler^®^ 480 System (Roche, Basel, Switzerland) with subsequent data analysis performed as per the manufacturers’ instructions and as described previously [3, 4]. Taking into consideration the poor quality of our samples, the recommended ∆C_q_ threshold was relaxed so that samples that had ∆C_q_ values of ≤6 (instead of the recommended 5) were further processed [3, 4].

### Sodium bisulfite conversion

FFPE tumour-enriched DNA samples (16–750 ng) were bisulfite converted using the EZ DNA Methylation-Gold kit (Zymo, Irvine, CA, USA), and eluted in a final volume of 8 μl elution buffer.

### Restoration of bisulfite converted FFPE tumour-enriched DNA

Restoration of bisulfite converted FFPE tumour-enriched DNA was performed using the Infinium HD FFPE Restore kit (Illumina, San Diego, CA, USA) as per the manufacturer’s instructions and as previously described [3, 4] except that restored bisulfite converted samples were eluted in a final volume of 10 ul nuclease free water.

### QC checkpoint 3: bisulfite-specific qPCR assay

The presence of FFPE tumour-enriched DNA after bisulfite conversion and restoration was determined using an in-house qPCR assay. Primers specific for bisulfite converted DNA were designed using EpiDesigner (http://www.epidesigner.com) (forward sequence: 5′ tAA GGT AtA ATt AGA GGA TGG GAG GGA t; reverse sequence: 5′ aaC AAA CTC Aaa TAa AAT TCT TCC TC) to amplify a 134 bp region (hg19:chr17:41277493-41277626) within the promoter of the *breast cancer 1, early onset* (*BRCA1*) gene (GenBank: L78833.1) [9]. Lower-case letters correspond to converted cytosines. The amplicon sequence is as follows: 5′ tAA GGT AtA ATt AGA GGA TGG GAG GGA tAG AAA GAG CCA AG**C G**TC TCT **CG**G GGC TCT GGA TTG GCC ACC CAG TCT GCC CC**C G**GA TGA **CG**T AAA AGG AAA GAG A**CG** GAA GAG GAA GAA TTt TAt tTG AGT TTG tt. CpG dinucleotides are in bold. The qPCR primers and the PCR conditions were developed to ensure that products be unaffected by the methylation status of the template (data not shown) [10].

Each reaction consisted of 1X SYBR Green I Master (Roche, Basel, Switzerland), 300 pM each of forward and reverse primers (Integrated DNA technologies, Coralville, IA), and 3 ul diluted restored bisulfite converted FFPE tumour-enriched DNA (diluted 1:3 in nuclease free water). The reaction was equilibrated to 10 μl with nuclease free water (Life Technologies, Carlsbad, CA, USA).

QPCR cycling conditions were as follows: initial polymerase activation for 5 min at 95 °C followed by 40 cycles of DNA denaturation for 10 s at 95 °C, primer annealing for 30 s at 60 °C and extension for 90 s at 72 °C. Subsequent melting of the amplified product was performed from 97 °C to 65 °C for 60 s. Fluorescent data was acquired on the green channel.

All samples were assayed in duplicate and non–bisulfite converted, unrestored U266 multiple myeloma cell line DNA was used as a negative control. Subsequent data analysis was performed similar to QC checkpoint 2 except that the difference in C_q_ value (ΔC_q_) was determined by subtracting the average C_q_ value of each FFPE tumour-enriched DNA sample from the average C_q_ value of the negative control (ΔC_q=_ Average C_qNegative control_ − Average C_qTumour-enriched DNA_). Using the Infinium-recommended ΔC_q_ threshold for QC checkpoint 2 as a reference (C_q_ difference of 5) and taking into account the poor quality and limited quantity of FFPE tumour-enriched DNA, a more relaxed ΔC_q_ threshold for this QC checkpoint was adopted. Only restored bisulfite converted FFPE tumour-enriched DNA with a ΔC_q_ value of ≥4 was assayed on the HM450K platform.

### Infinium HumanMethylation450 Beadchip assay

The Infinium HumanMethylation450 Beadchip assay (Illumina, San Diego, CA, USA) was performed as per manufacturer’s instructions specific for formalin-fixed embedded-material. The following replicates were included for every 48 samples to ensure the reproducibility of data: (1) one technical replicate from good quality bisulfite converted cell line DNA (U266 multiple myeloma cell line) to test for possible batch effects between different reagents and beadchips used, and different processing times and (2) one technical replicate from restored bisulfite converted FFPE tumour-enriched DNA, where each replicate was placed on a different beadchip to test for possible chip effects between beadchips.

### Data analysis

Data from samples assayed on the HM450K platform were imported into the R environment (R Programming Software version 2.15.1) as previously described [2] and processed using the *Minfi* package version 1.10.1 [11]. The following criteria were applied to evaluate the overall data quality and performance of individual samples across all probes and individual probes within each sample, respectively: (1) average detection p-value across all probes of p ≤ 0.01; (2) individual probe detection p-value of p ≤ 0.05. Individual FFPE tumour-enriched DNA or probes that failed either criterion were removed from further analysis. Any sample with more than 1 % failed probes from a total of 485,512 probes were also classed as “failed” and excluded for the purposes of this analysis.

### Analysis of TCGA HM450K methylation data

Subtype information and associated level 3 methylation signals (calculated beta values mapped to the genome) from 156 breast tumours were obtained from the TCGA Download Portal (https://tcga-data.nci.nih.gov/tcga/tcgaHome2.jsp) [12]. The data consisted of the following subtypes based on the expression of 50 genes and classified using the prediction analysis of microarray (PAM): 86 Luminal A, 39 Luminal B, 22 basal-like and 9 HER2-enriched [13]. The mean beta value, standard deviation and 95 % confidence interval (CI) were calculated for each of the 48 methylation probes specific to the *BRCA1* gene (5 additional probes were classified as ‘NA’ due to having detection p-values >0.05) across all tumours of the same subtype, resulting in 3 values per methylation probe for each subtype.

## Results

**Fig. 2:**
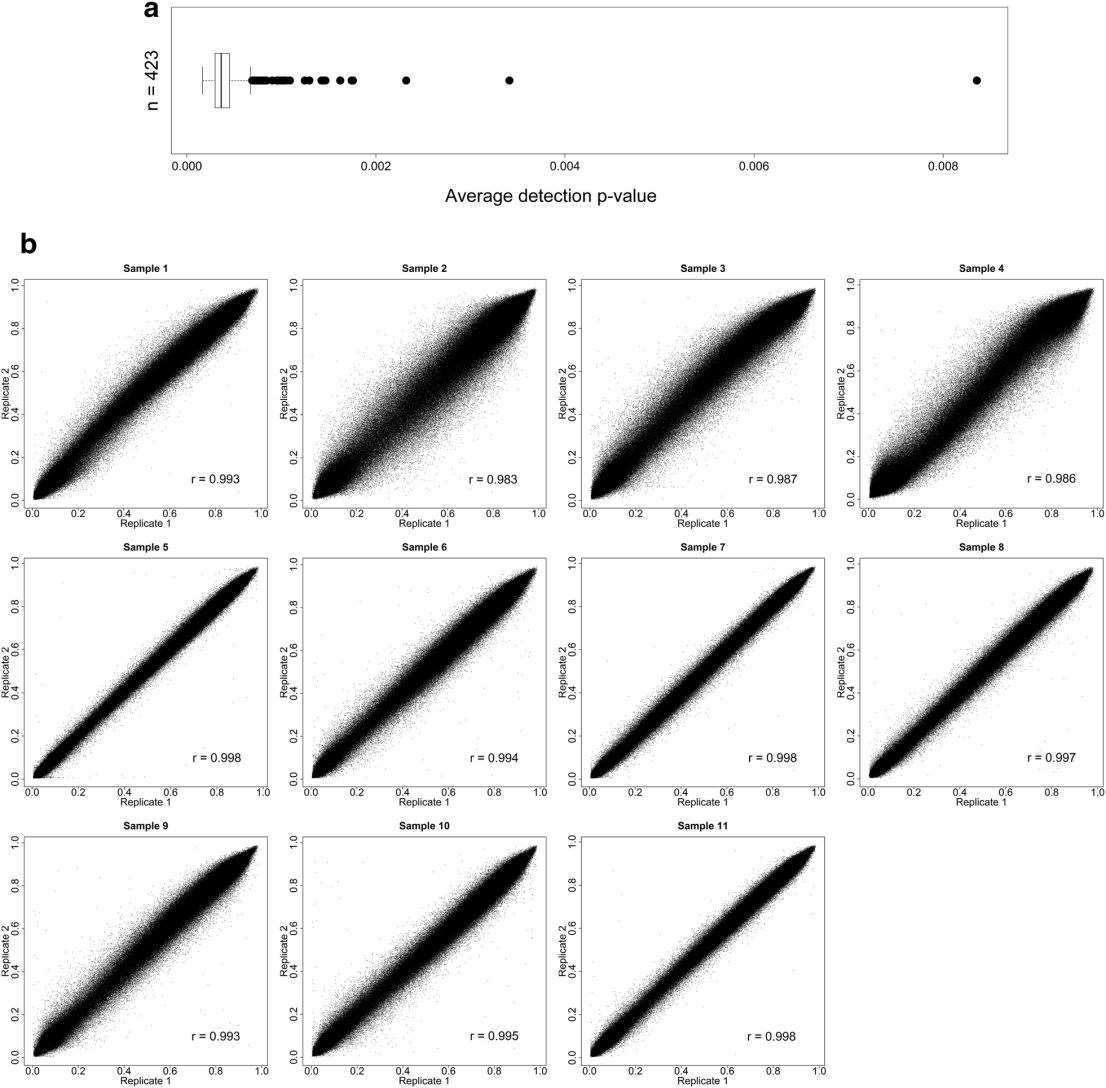
Post HM450K assay data quality checks of FFPE tumour-enriched DNA. **a** Average probe detection p-values across all probes for each sample. Each *black dot* represents a single sample. The average detection p–value across all probes for all samples (n = 423) was 4.54 × 10^−4^. **b** Scatterplots of replicates assayed on different beadchips (n = 11). The average correlation coefficient (r_ave_) across all replicates was 0.993

DNA was macrodissected from marked up areas of 474 FFPE breast tumours. Five samples did not have a detectable amount of DNA at QC checkpoint 1. Of the remaining 469 FFPE tumour-enriched DNA samples that were progressed past QC checkpoint 2, 42 (8.93 %) samples failed to progress past QC checkpoint 3 with ∆Cq values ranging from 0 (no amplification) to 3.77. The methylome of 427 FFPE tumour-enriched DNA were subsequently evaluated on the HM450K platform (Fig. 1). Of these, 4 (<1 %) had an average detection p-value across all probes of ≥0.01 and were removed from further analysis. Based on our criteria, 5 % (24,816) of probes were removed, with 460,696 common probes remaining across 423 FFPE tumour-enriched DNA for downstream data analysis. The average detection p-value across these probes for all remaining samples (n = 423) was 4.54 × 10^−4^ (Fig. 2a) with high correlations observed between sample replicates (correlation coefficients r > 0.98) (Fig. 2b) thereby confirming the capacity of our protocol to generate reproducible, high quality data.

We further evaluated the 4 failed samples on the HM450K platform and 16 randomly selected ‘successful’ samples at the QC checkpoints (Table 1). We did not observe a correlation between age of tumour material (8–20 years), performance at any of the QC checkpoints and subsequent performance on the HM450K platform.

**Table 1:** Age of tumour material, performance at QC checkpoints and number of failed HM450K probes in failed and a subset of successful samples on HM450K platform

Sample	Age of tumour material (years)	QC 1 (total ng)	QC 2 (∆C_q_ ≤ 6)	QC 3 (∆C_q_ ≥ 4)	Number of failed probes
2657^a^	20	209	1.43	5.45	125,491
3282^a^	20	483	0.68	8.09	196,914
25098^a^	11	334	3.25	5.94	28,751
27042^a^	7	671	1.83	9.84	22,334
1537	15	47	0.86	5.56	502
2245	12	256	1.04	8.16	272
6075	19	27.3	1.00	4.28	788
6112	20	159.8	1.61	6.72	534
8613	18	51.5	1.87	4.64	794
10350	13	1078	2.7	4.83	9995
10780	14	749	4.1	4.24	6678
15291	14	114.6	1.24	6.96	485
17940	17	583	4.13	4.83	5436
21200	10	71.2	1.17	4.17	694
21635	13	172.4	2.73	4.62	1083
35915	9	305	2.78	7.28	512
35995	8	270	4.28	4.00	3379
37246	12	180.5	3.74	4.14	2714
40593	12	63.9	1.64	6.04	1079
41223	11	1579	2.95	6.42	1489

^a^Denotes failed samples on the HM450K assay (according to study criteria)

### Performance of internal control probes

We assessed the performance of sample-specific and sample-independent internal control probes present on the HM450K beadchip (http://www.illumina.com) between ‘failed’ and ‘successful’ tumour-enriched DNA samples and two unrestored U266 cell line genomic DNA (Additional file 1: Figure S1, Additional file 2: Figure S2). The performance of the controls probes was variable between tumour-enriched samples. The majority of the control probes for samples 2657 and 3282 which had the largest number of failed HM450K probes performed poorly and had the highest level of background signal amongst the 22 samples evaluated.

### Methylation at the *BRCA1* gene

**Fig. 3:**
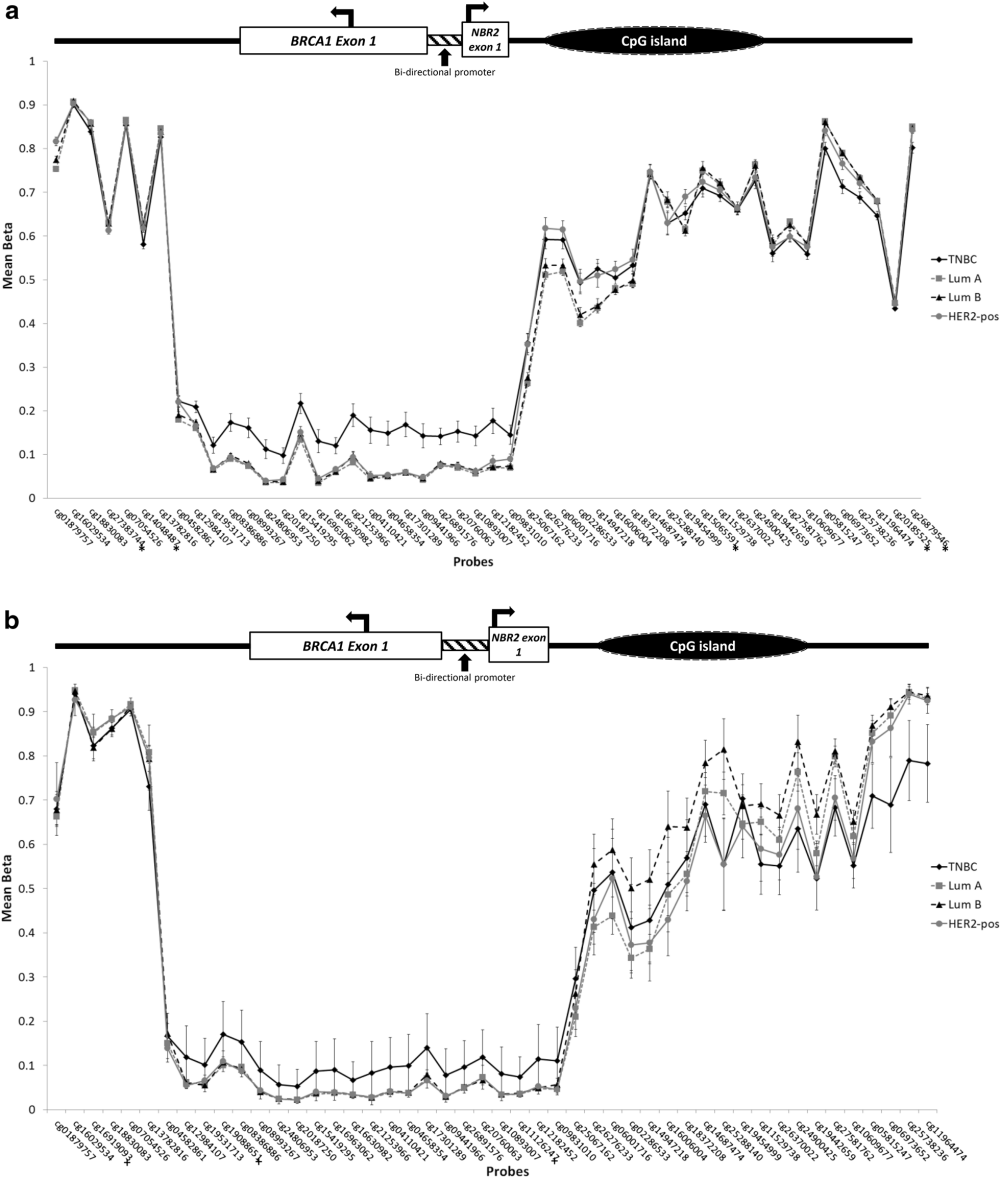
Probe mean beta values at the *BRCA1* gene. **a** Mean beta values measured from 419 tumour-enriched DNA in our dataset. Twenty probes overlapping exon 1 of *BRCA1*, exon 1 of *NBR2* and their shared bi-directional promoter showed increased methylation in triple-negative breast cancers (TNBC) compared with Luminal A (Lum A), Luminal B (Lum B) and HER2-positive (HER2-pos) subtypes (* denotes probes not present in the TCGA dataset). **b** Mean beta values measured from 156 tumour-enriched DNA in TCGA. Twenty-two probes overlapping exon 1 of *BRCA1*, exon 1 of *NBR2* and their shared bi-directional promoter showed increased methylation in basal-like breast cancers (TNBC) compared with Luminal A (Lum A), Luminal B (Lum B) and HER2-enriched (HER2-pos) subtypes (+ denotes probes not present in our dataset)

The HM450K assay measures methylation at 53 CpG sites (53 methylation probes) across the *BRCA1* gene. Three probes (cg19088651, cg11126247 and cg16919093) did not meet the QC criteria and were removed from the analysis. The 423 remaining FFPE tumour-enriched breast DNA in our study consisted of the following subtypes: 238 Luminal A, 87 Luminal B, 63 triple-negative and 30 HER2-positive. The mean beta value, standard deviation and 95 % CI for each *BRCA1*-specific probe across each subtype were calculated and plotted as a line graph. Approximately 20 probes along the *BRCA1* promoter region displayed higher mean beta levels in the triple-negative breast tumours compared to other subtypes (student’s t-test, p < 10^−5^) (Fig. 3a). This probe set overlapped *BRCA1* exon 1, exon 1 of its’ neighbouring gene, *NBR2* [14] and their shared bi-directional promoter [15].

We sought to compare our results against publicly available data from The Cancer Genome Atlas where genome-wide methylation was measured using the same platform and on DNA extracted from fresh frozen material thereby representing good quality DNA [12]. We compared methylation signals at *BRCA1* between the basal-like, Luminal A, Luminal B and HER2-enriched breast tumour subtypes and although not at a significant level, we observed a similar trend along the same 20 probes where increased methylation was observed specifically in basal-like breast tumours compared with other subtypes (Fig. 3b). An additional two probes within the same region of *BRCA1* which failed in our dataset also displayed the same trend.

## Discussion

This protocol facilitated the successful application of the HM450K platform to DNA extracted from FFPE tumour material and is relevant for any large population-based translational epigenetic studies of human disease. This assertion is based on assessment of the quality metrics and consistency of the data with prior research data examining methylation at the *BRCA1* promoter.

Methylation of the *BRCA1* promoter along with the corresponding loss of *BRCA1* expression in mutation-negative breast cancer is well described for both familial and sporadic breast cancer and has been demonstrated using low-throughput loci-specific methods [16–18]. Promoter methylation of this gene has also been associated with the triple-negative breast cancer subtype and/or histological features commonly associated with triple-negative breast cancers [19–22].

In our data 20 probes along the *BRCA1* promoter region displayed higher mean beta levels in the triple-negative breast tumours compared to other subtypes. This region encompassed a region of the *BRCA1* promoter (CpG sites −44 to +55 relative to the transcription start site and corresponding to the region between probes cg19531713 and cg17301289) that has been specifically measured by MethyLight Real-time PCR in previous studies [20].

The modified protocol described here incorporates an important third QC checkpoint. Assuming all samples that failed the QC would have also failed the HM450K assay, incorporation of this checkpoint increased the success rate of our samples on the HM450K platform from 90 % to the reported 99 %. Using this modified protocol, we were able to identify and remove samples that were likely to fail prior to application on the HM450K platform.

However, there are several aspects to this data that are important to consider further. Firstly, as we excluded samples that fell below the described threshold (ΔC_q_ value ≥ 4) we do not have data that confirms that these samples would not have generated data, on the HM450K platform, that met our criterion for success (average detection p-value across all probes of p ≤ 0.01 and individual probe detection p-value of p ≤ 0.05). The costs associated with running this platform makes this challenging to test, but data from 2 archival FFPE tumour samples which failed QC checkpoint 3 (ΔC_q_ 2.52 and 0) in a subsequent study in our laboratory produced very poor quality data with 61,803 and 200,552 failed probes, respectively [data not shown]. This finding supports our protocol to exclude samples with ΔC_q_ value ≤4 at QC checkpoint 3.

Secondly, the *BRCA1* promoter amplicon utilised in QC checkpoint 3 includes CpGs sites. Although our methodology was developed to balance amplification of both the methylated and non-methylated template [10], this is difficult to achieve due to potential PCR bias favouring the amplification of the unmethylated template [23]. Our assay was not designed to differentiate between methylated and unmethylated template but, if this is important to other related applications, it would be more appropriate to select an alternate amplicon (without CpG sites) that would offer a superior assay.

We are also aware that we cannot exclude the possibility of Loss of Heterozygosity or homozygous deletion at the BRCA1 locus being responsible for a proportion of the QC checkpoint failures. Further investigation using other molecular techniques such as Multiplex Ligation-dependent Probe Analysis (MLPA) and Sanger sequencing of heterozygous SNPs flanking this region is certainly warranted but not within the scope of this technical report.

The performance of the HM450K internal control probes did not always correlate with the detection p-values. For instance, the internal control probes for sample 27042 performed satisfactorily although having a high number of probes with detection p-values >0.05. In contrast, sample 35995 passed all our data QC hurdles although some of the bisulfite conversion II control probes showed higher background signal intensities.

This protocol is suitable for the analysis of macrodissected FFPE tumour-enriched DNA samples of variable quantities and quality and can be reliably and systematically applied to hundreds of samples in a standard and controlled manner. Moreover, it has the potential to be translated into molecular pathology diagnostic services as the assessment of methylation becomes more clinically applied in the diagnosis and treatment of disease. This methodology will have utility in population-based translational epigenetic studies and is applicable to a wide variety of translated studies interested in analysis of methylation and its role in the predisposition to disease and disease progression.

## Conclusions

This protocol facilitated the successful application of the HM450K platform to DNA extracted from archival FFPE tumour material and is relevant any large population-based translational epigenetic studies of human disease.

## Additional files

10.1186/s13104-015-1487-z Performance of HM450K sample-independent control probes for failed and a subset of successful samples. Sample-independent control probes measured the staining (A), extension (B), target removal (C) and hybridisation (D) steps in the HM450K assay on the red and green channels. Failed samples are boxed.
10.1186/s13104-015-1487-z Performance of HM450K sample-dependent control probes for failed and a subset of successful samples. Sample-dependent control probes measured background signal levels (negative; A), efficiency of bisulfite conversion using Infinium type I (B) and type II (C) probe designs, allele-specific extension of Infinium I and II probes (specificity; D) and restoration (E) and overall assay performance (non-polymorphic; F) on the red and green channels. Failed samples are boxed.

## Abbreviations

bp: base pair
*BRCA1*: *breast cancer 1, early onset*
CI: confidence interval
C_q_: quantitation cycle
DNA: deoxyribonucleic acid
FFPE: formalin-fixed paraffin-embedded
HM450K: Infinium HumanMethylation450 Beadchip
H&E: haematoxylin and eosin
HER2: *human epidermal growth factor receptor*
MCCS: Melbourne Collaborative Cohort Study
NBR2: *neighbour of BRCA1 gene 2*
qPCR: quantitative polymerase chain reaction
PAM: prediction analysis of microarray
QC: quality control
SNP: single nucleotide polymorphism
TCGA: The Cancer Genome Atlas Network
