# Supplementary figures and images for: Tools for translational epigenetic studies involving formalin-fixed paraffin-embedded human tissue: applying the Infinium HumanMethyation450 Beadchip assay to large population-based studies
Source: BMC Res Notes. 2015 Oct 6;8:543. doi: 10.1186/s13104-015-1487-z (PMC4595238; doi:10.1186/s13104-015-1487-z)

A) Staining

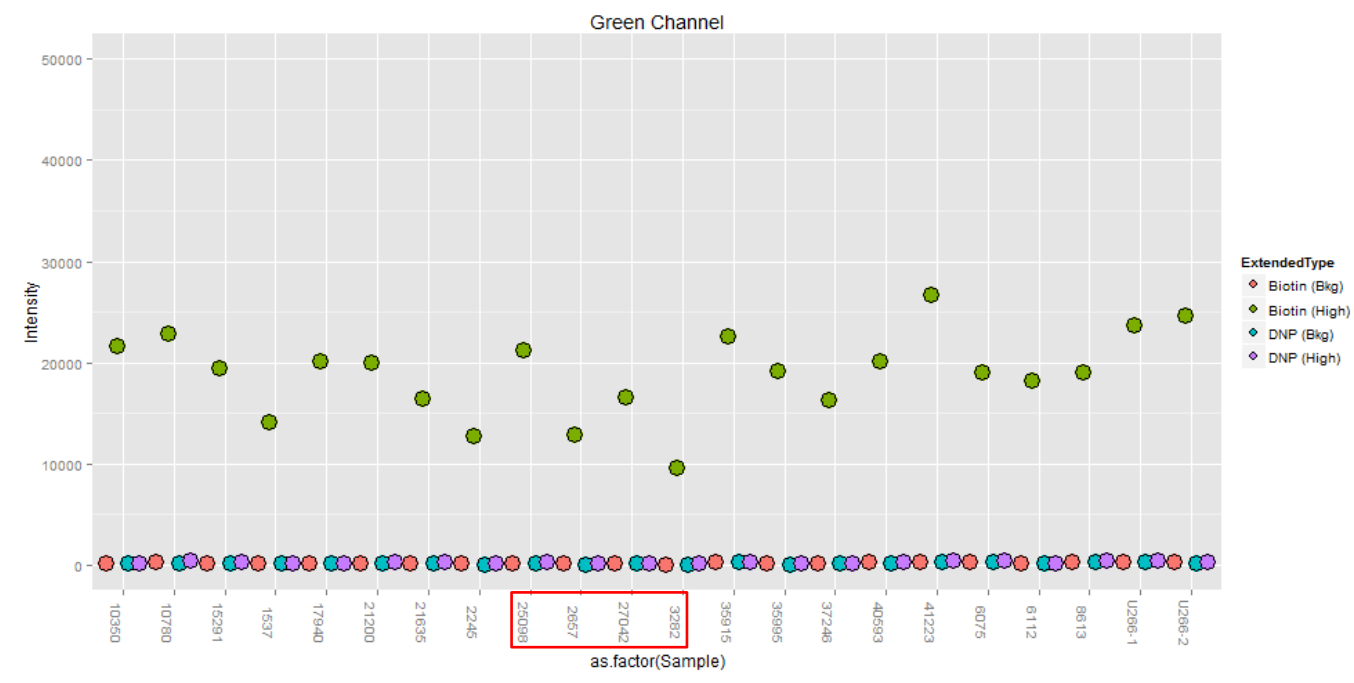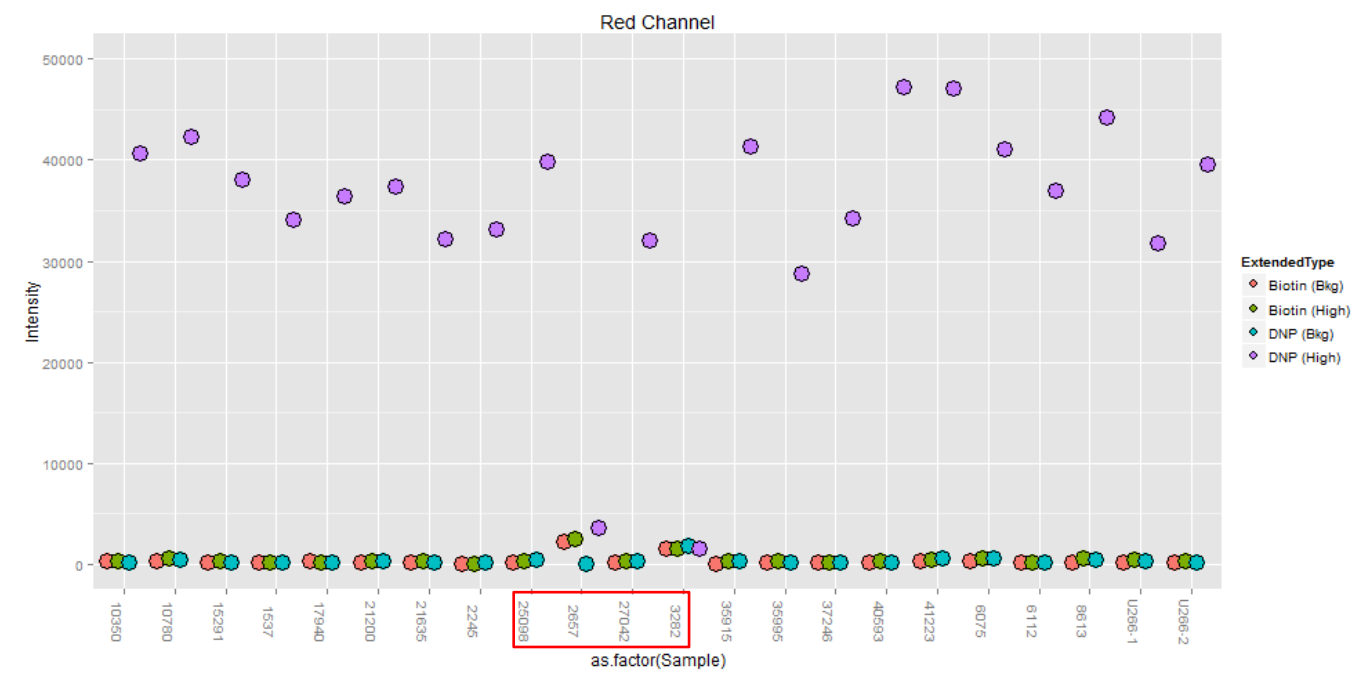

B) Extension

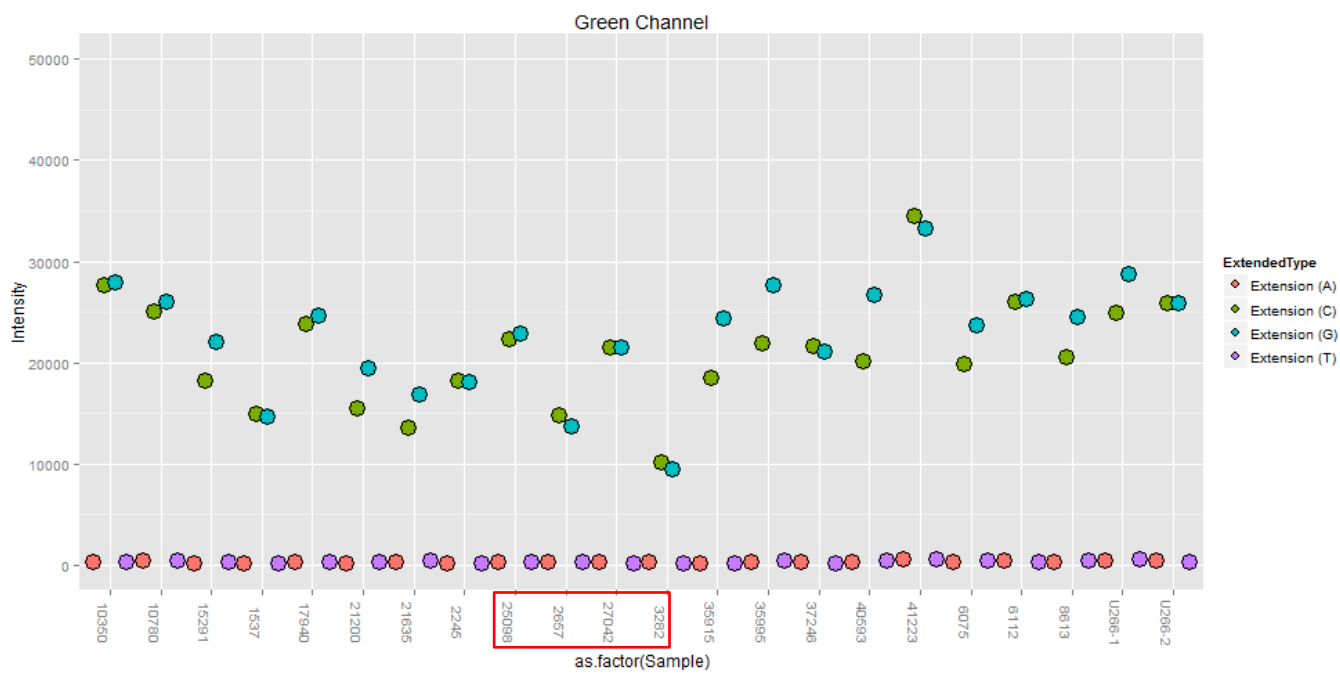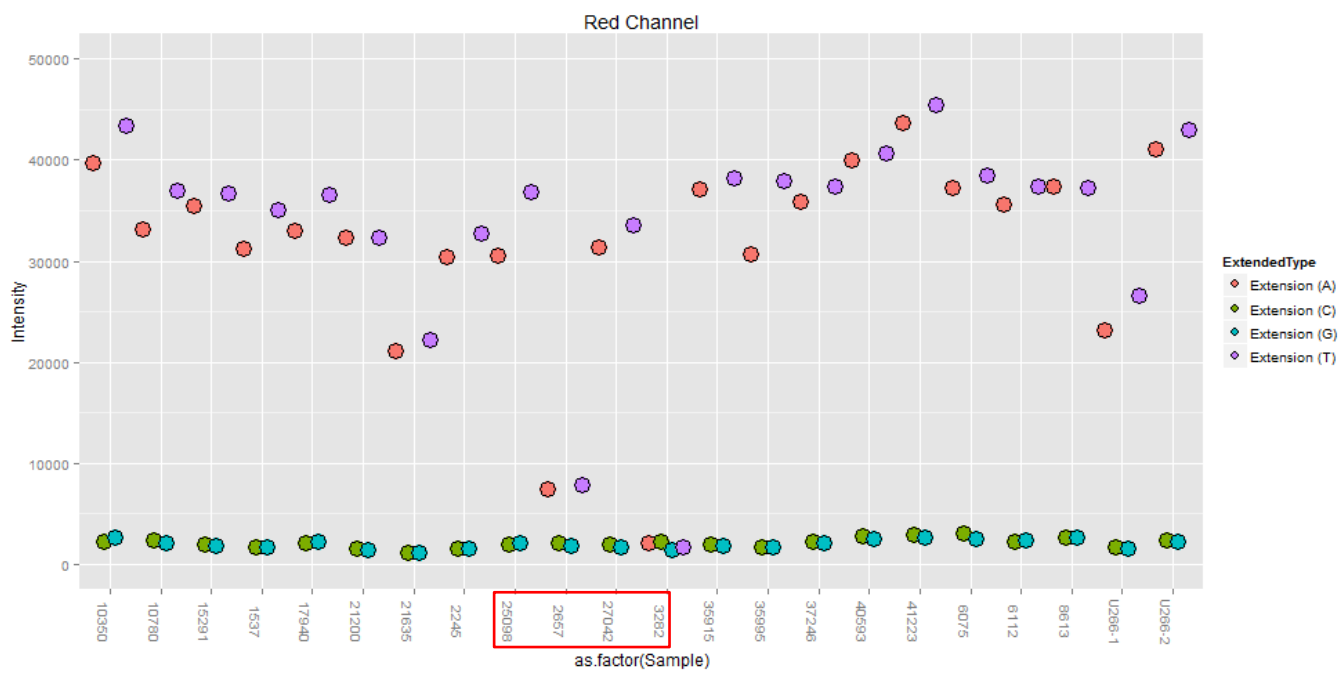

C) Target removal

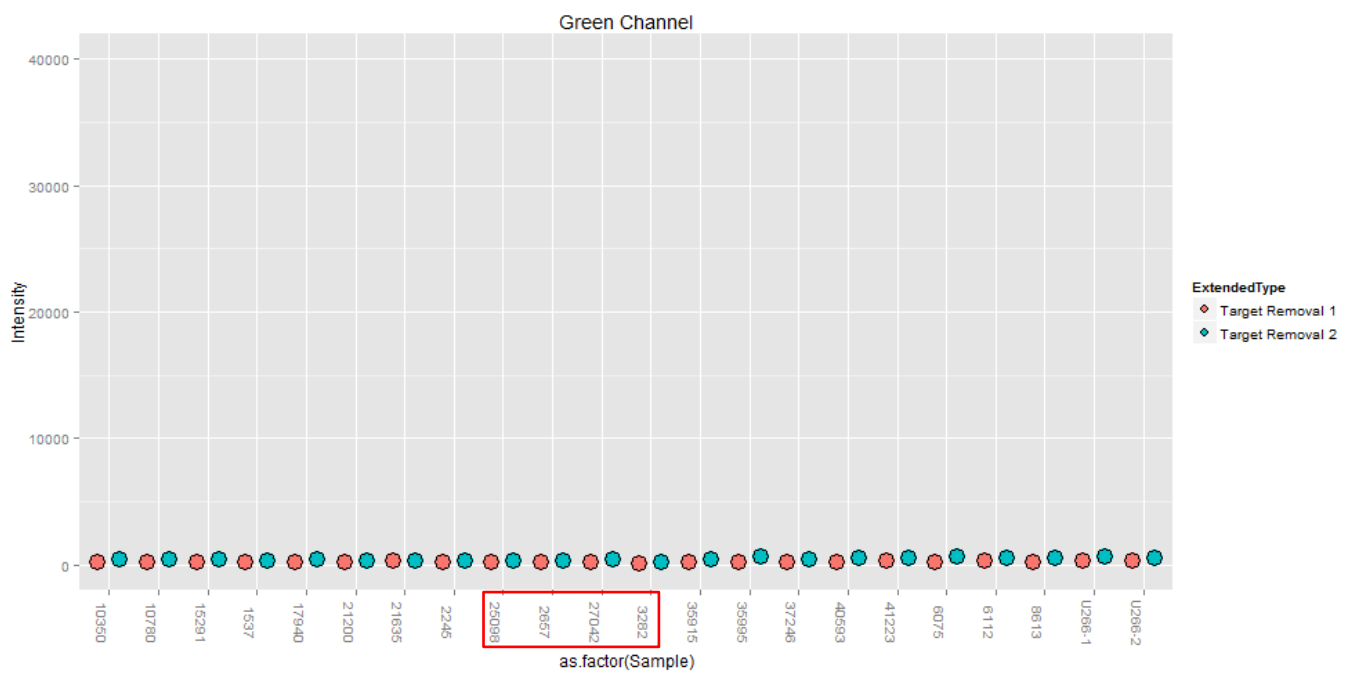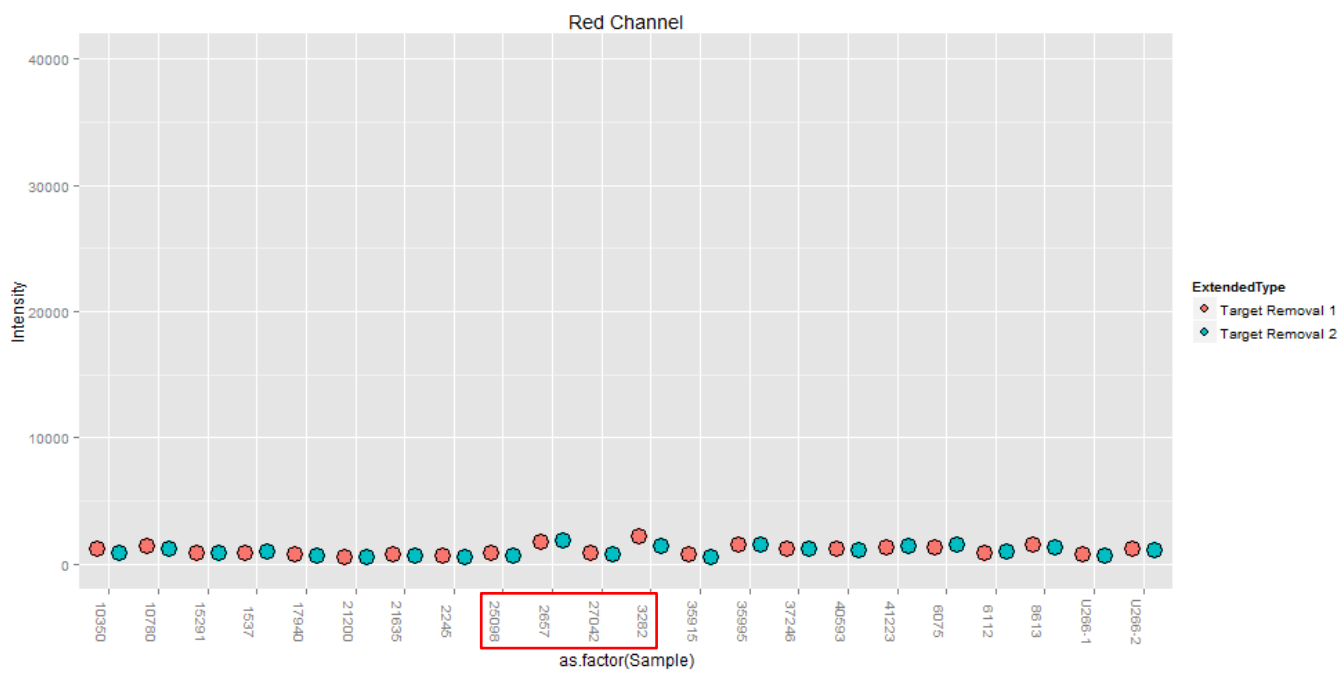

D) Hybridisation

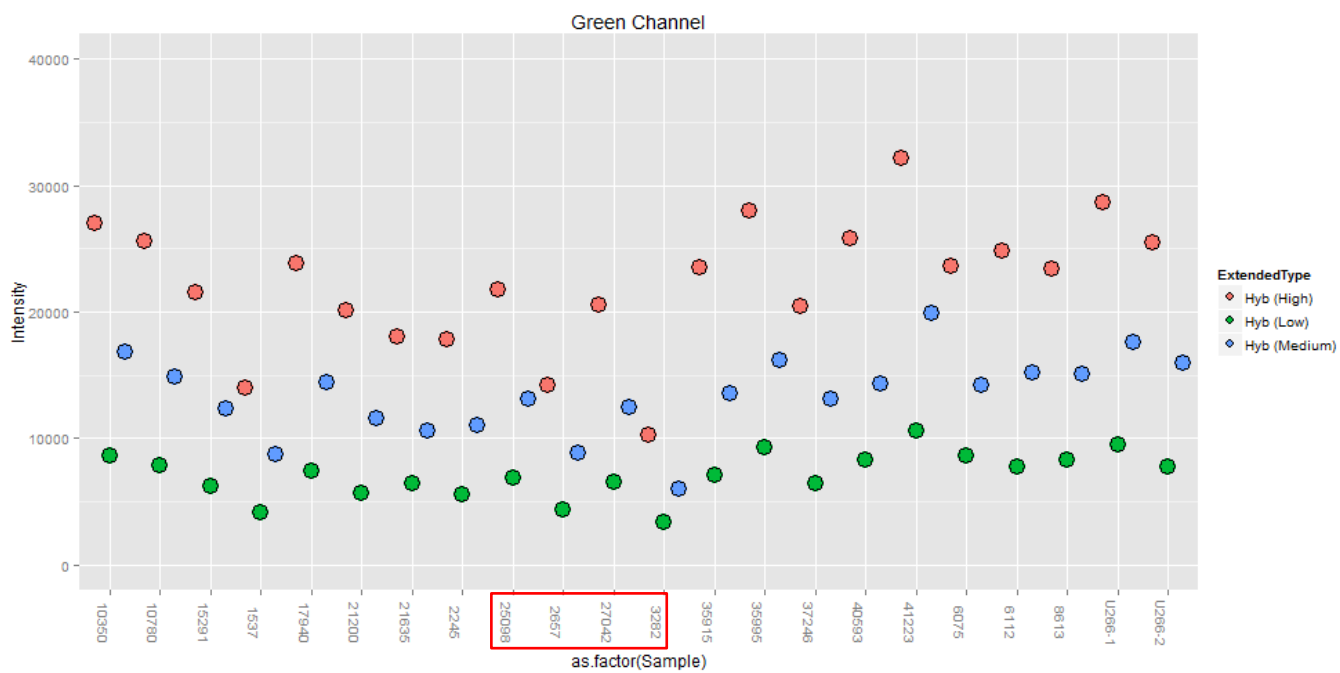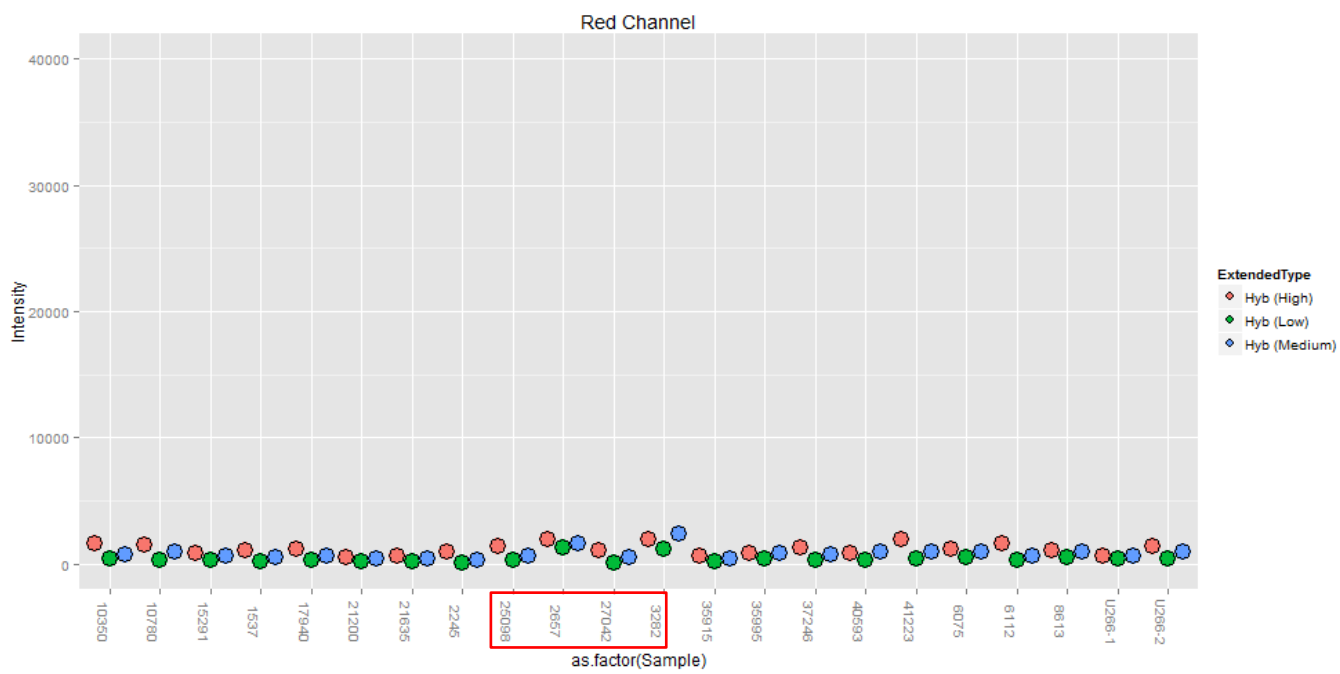

Supplement: Supplementary file 1 — 10.1186/s13104-015-1487-z Performance of HM450K sample-independent control probes for failed and a subset of successful samples. Sample-independent control probes measured the staining (A), extension (B), target removal (C) and hybridisation (D) steps in the HM450K assay on the red and green channels. Failed samples are boxed. [file 13104_2015_1487_MOESM1_ESM.pdf]
