# Supplementary material for: Tools for translational epigenetic studies involving formalin-fixed paraffin-embedded human tissue: applying the Infinium HumanMethyation450 Beadchip assay to large population-based studies
Source: BMC Res Notes. 2015 Oct 6;8:543. doi: 10.1186/s13104-015-1487-z (PMC4595238; doi:10.1186/s13104-015-1487-z)

A) Negative

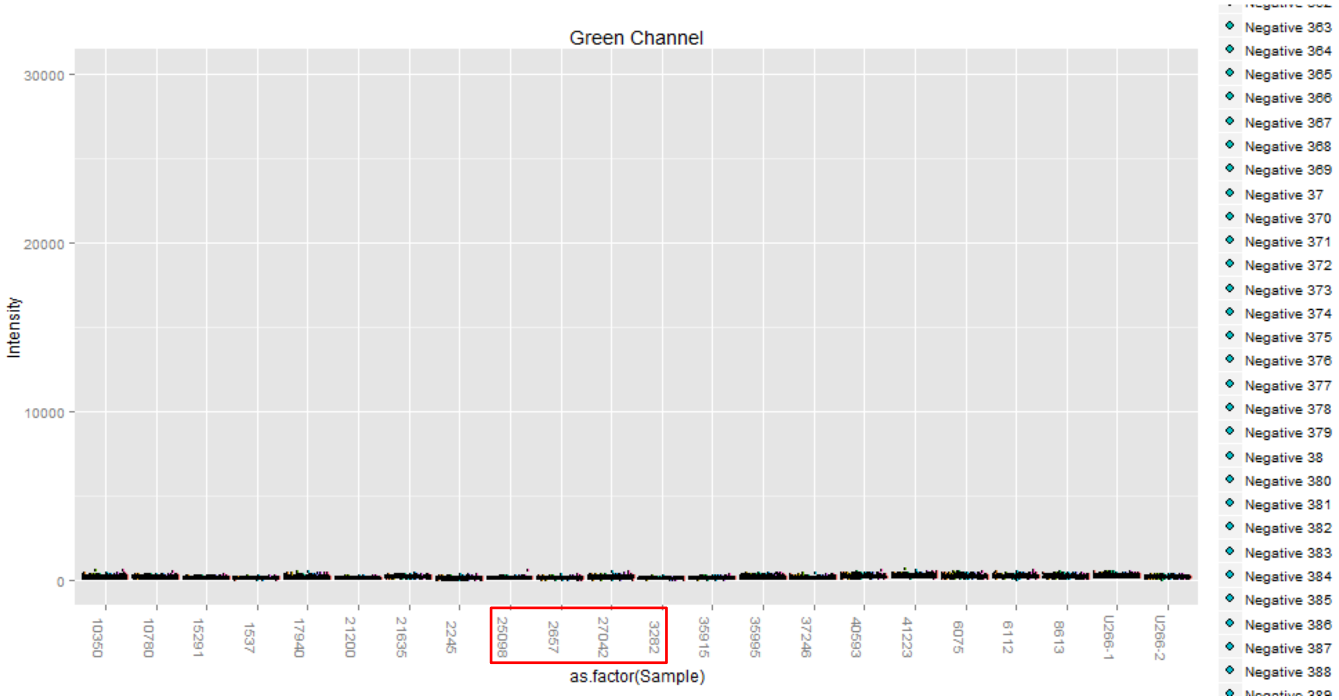
Green Channel
Intensity
30000
20000
10000
0
10350
10780
15291
1537
17940
21200
21635
2245
25098
2657
27042
3282
35915
35995
37246
40593
41223
6075
6112
8613
U266-1
U266-2
as.factor(Sample)
Negative 363
Negative 364
Negative 365
Negative 366
Negative 367
Negative 368
Negative 369
Negative 37
Negative 370
Negative 371
Negative 372
Negative 373
Negative 374
Negative 375
Negative 376
Negative 377
Negative 378
Negative 379
Negative 38
Negative 380
Negative 381
Negative 382
Negative 383
Negative 384
Negative 385
Negative 386
Negative 387
Negative 388

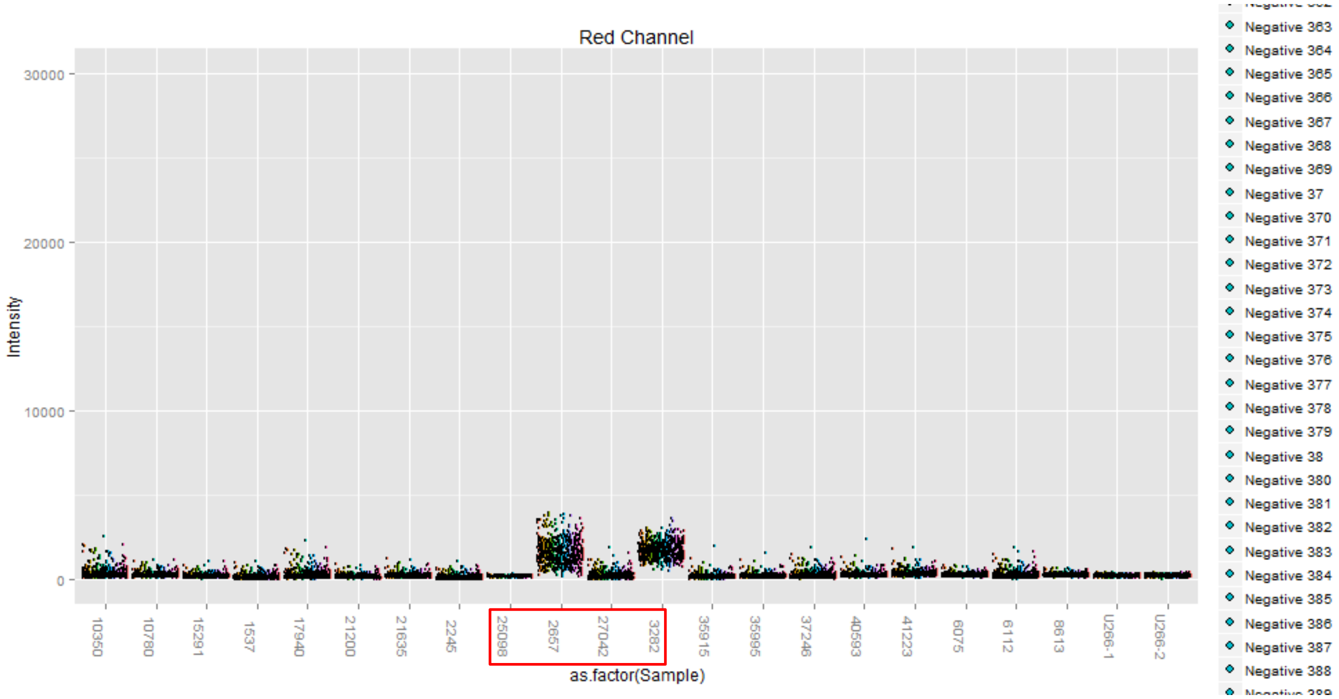
Red Channel
Intensity
30000
20000
10000
0
10350
10780
15291
1537
17940
21200
21635
2245
25098
2657
27042
3282
35915
35995
37246
40593
41223
6075
6112
8613
U266-1
U266-2
as.factor(Sample)
Negative 363
Negative 364
Negative 365
Negative 366
Negative 367
Negative 368
Negative 369
Negative 37
Negative 370
Negative 371
Negative 372
Negative 373
Negative 374
Negative 375
Negative 376
Negative 377
Negative 378
Negative 379
Negative 38
Negative 380
Negative 381
Negative 382
Negative 383
Negative 384
Negative 385
Negative 386
Negative 387
Negative 388

B) Bisulfite conversion I

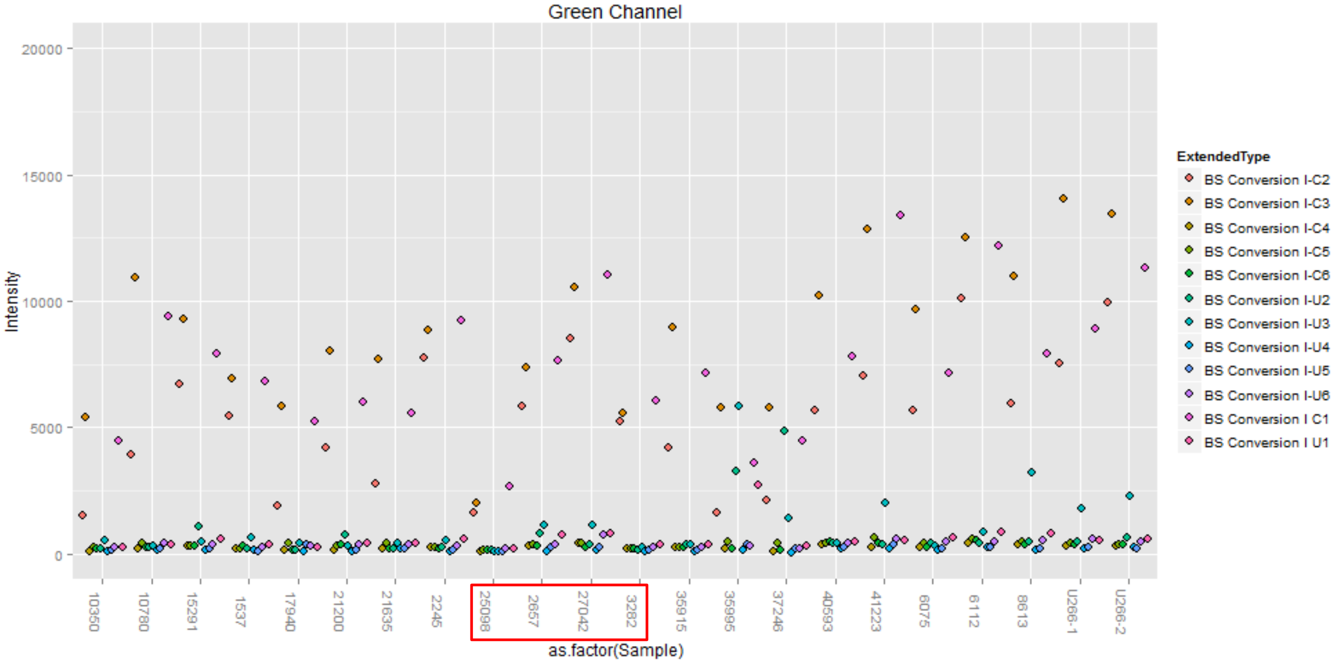

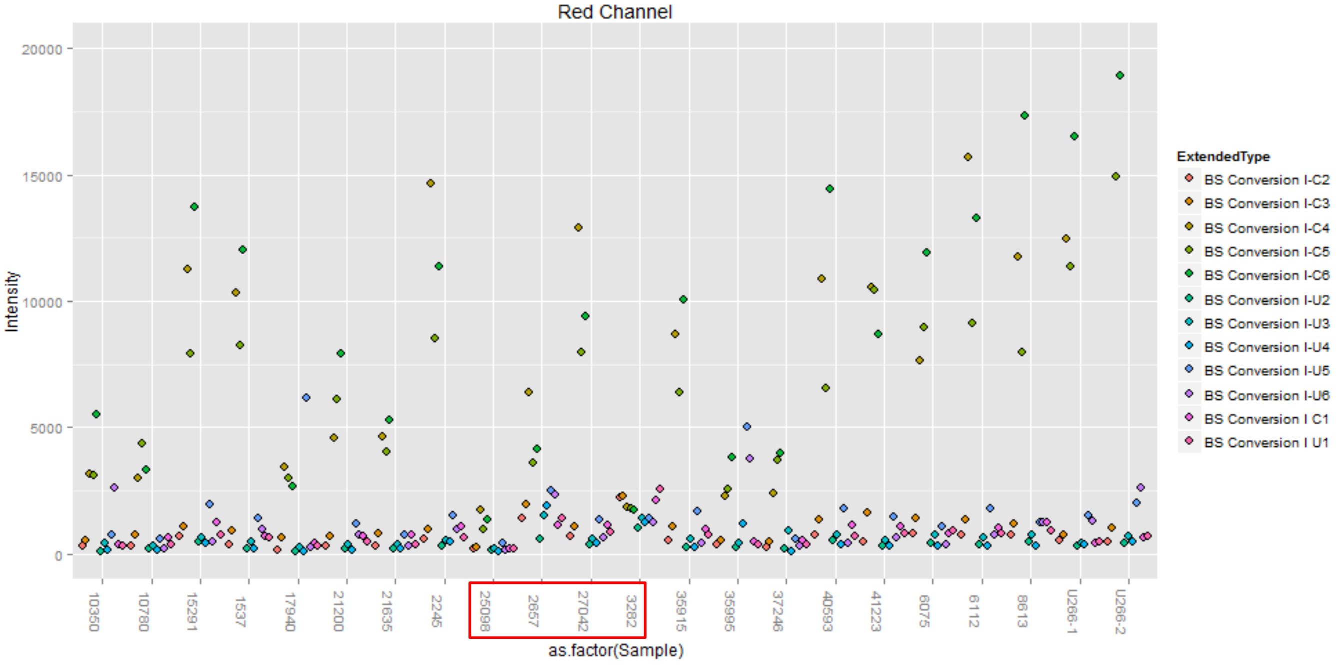

C) Bisulfite conversion II

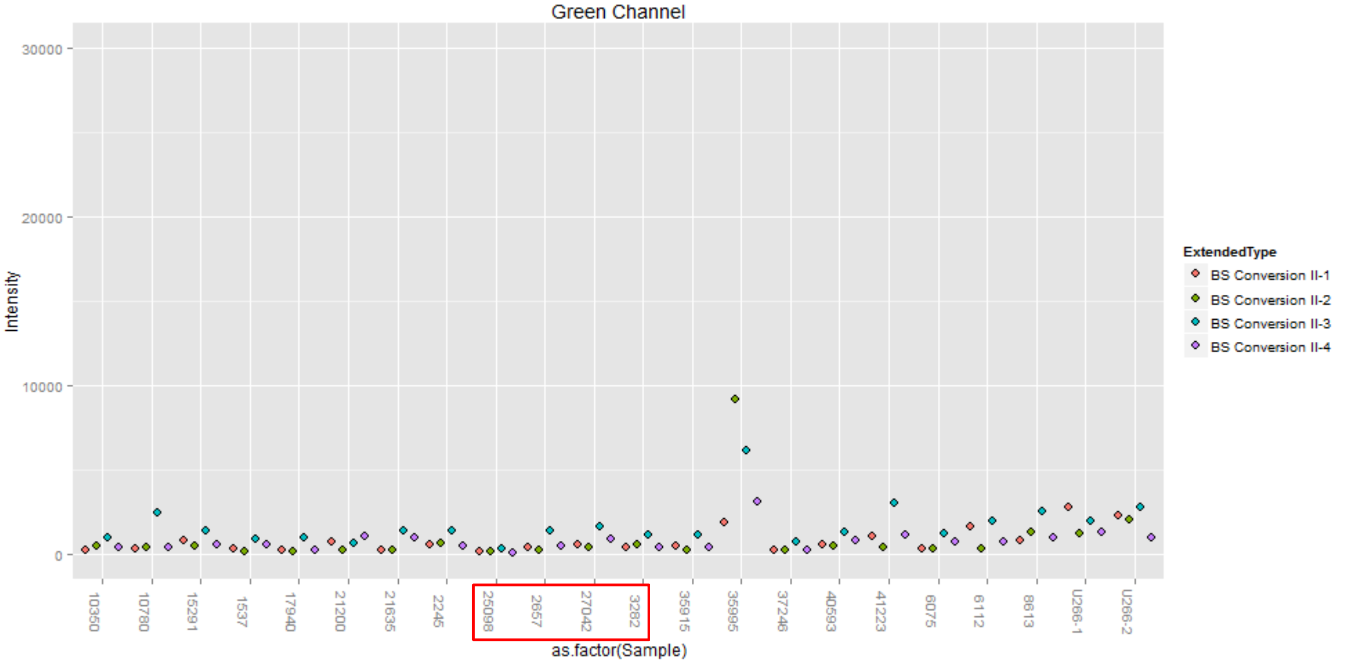

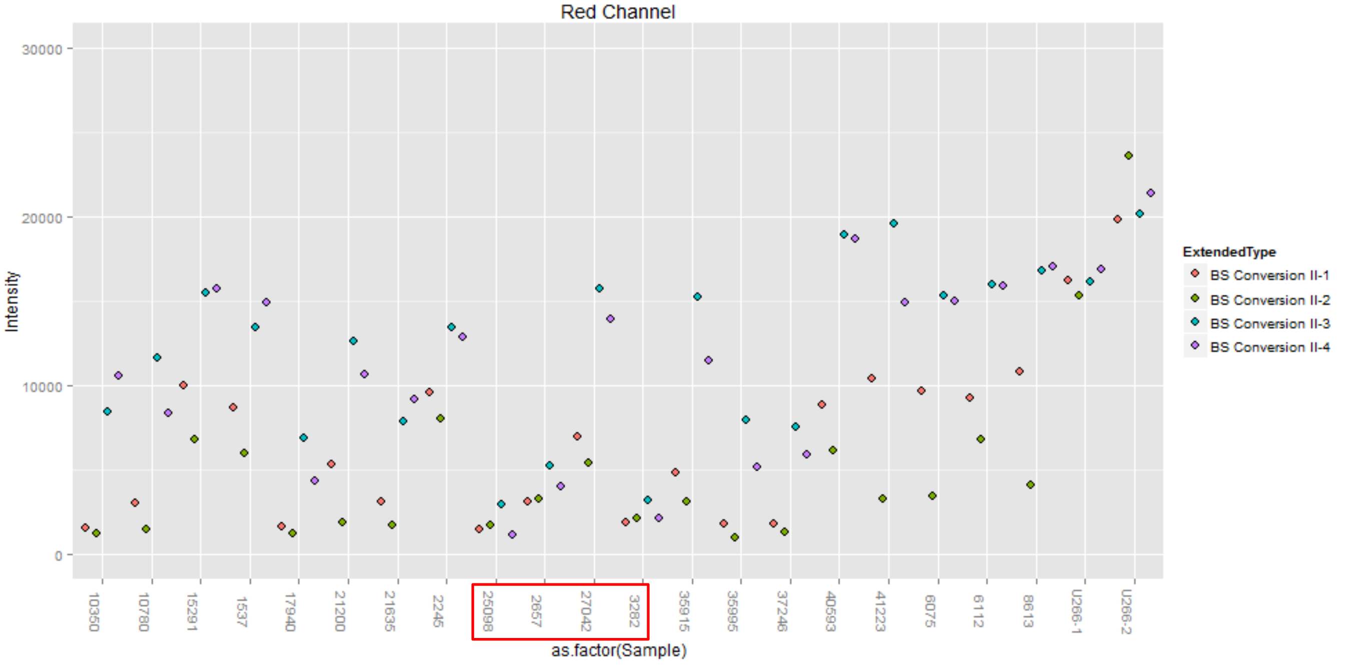

D) Specificity

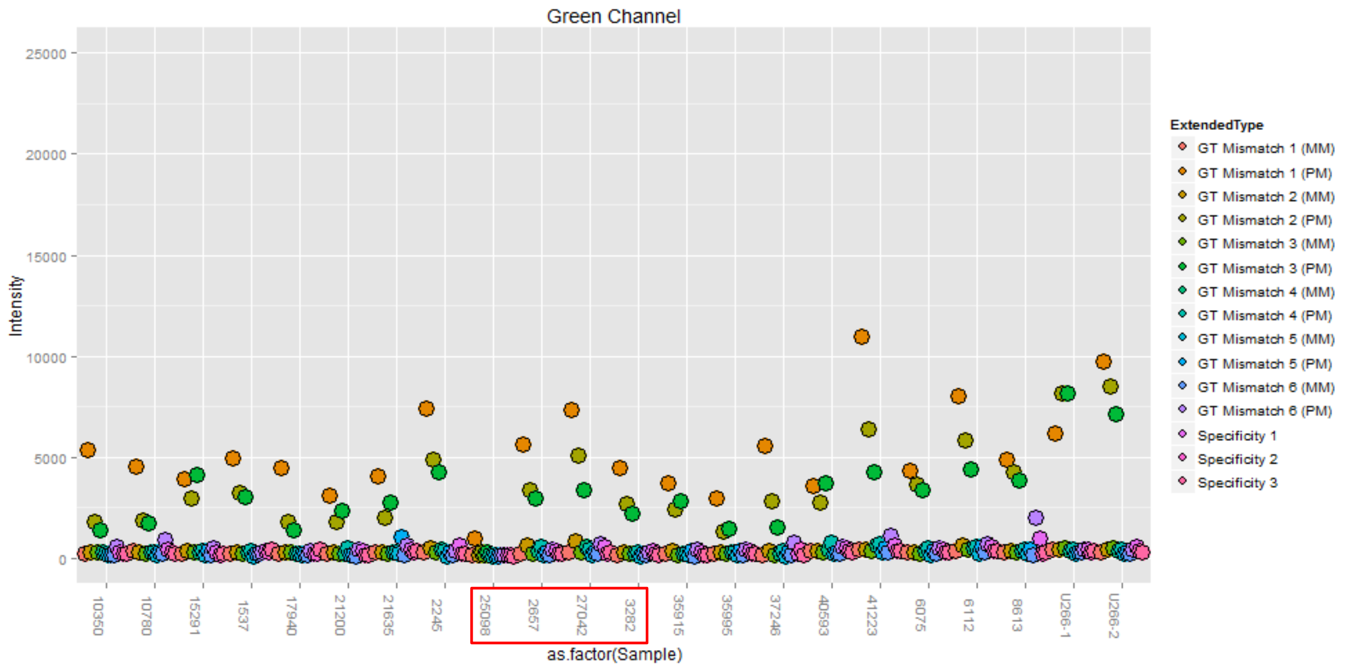

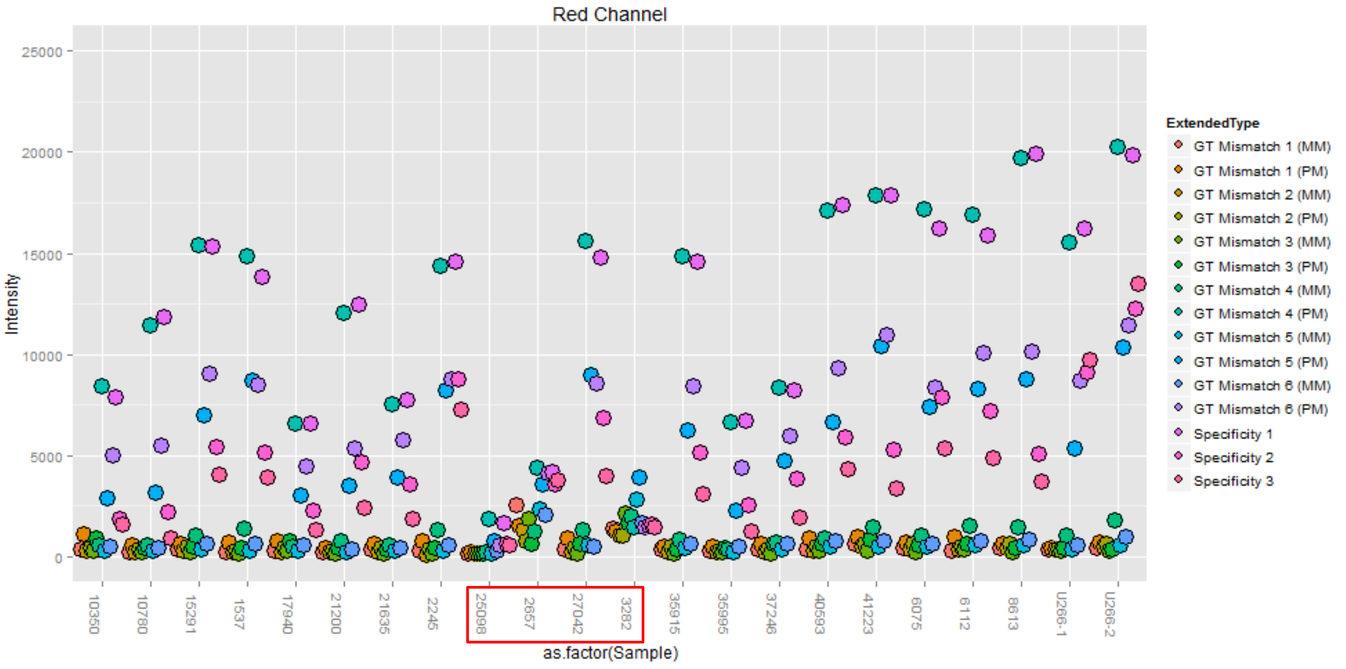

E) Restoration

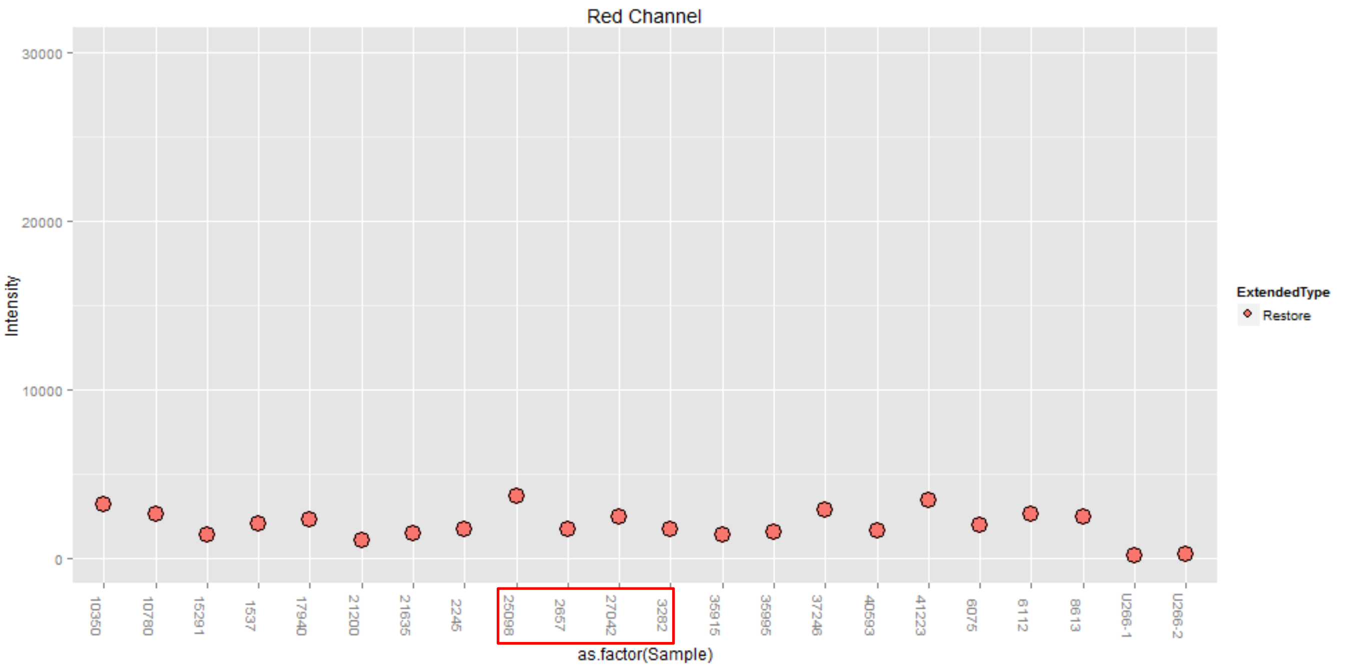

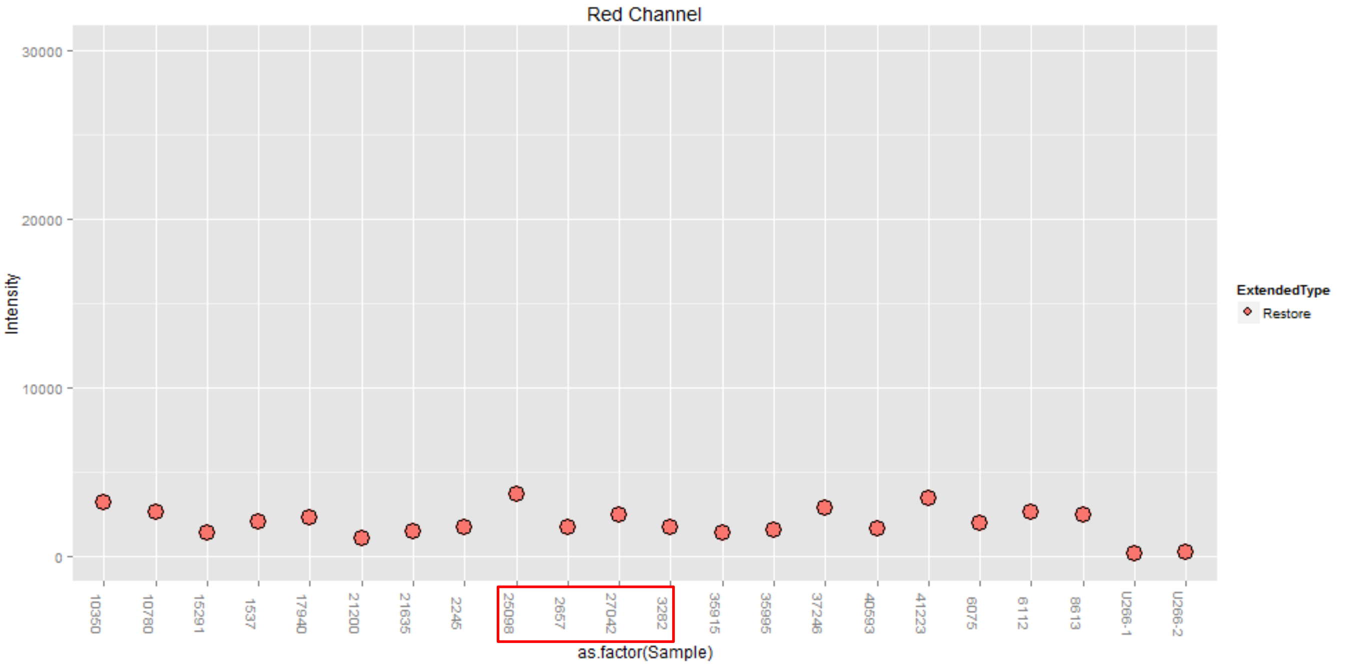

F) Non-polymorphic

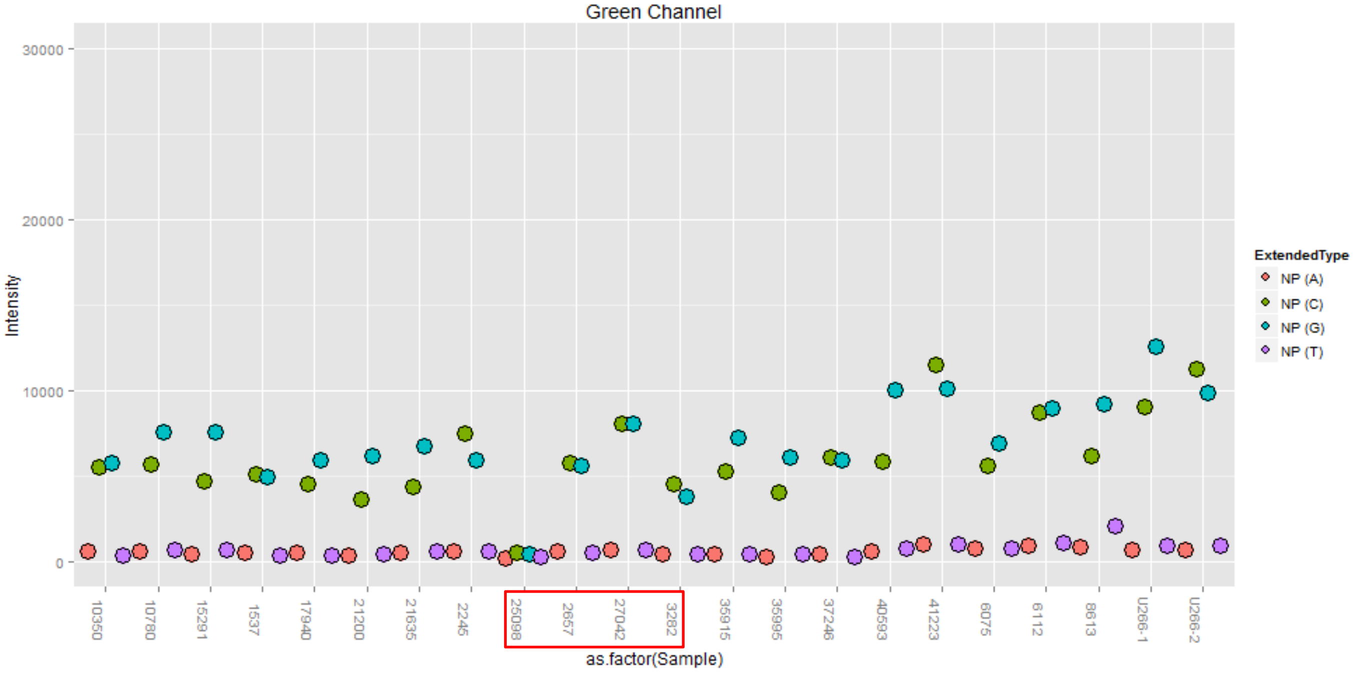

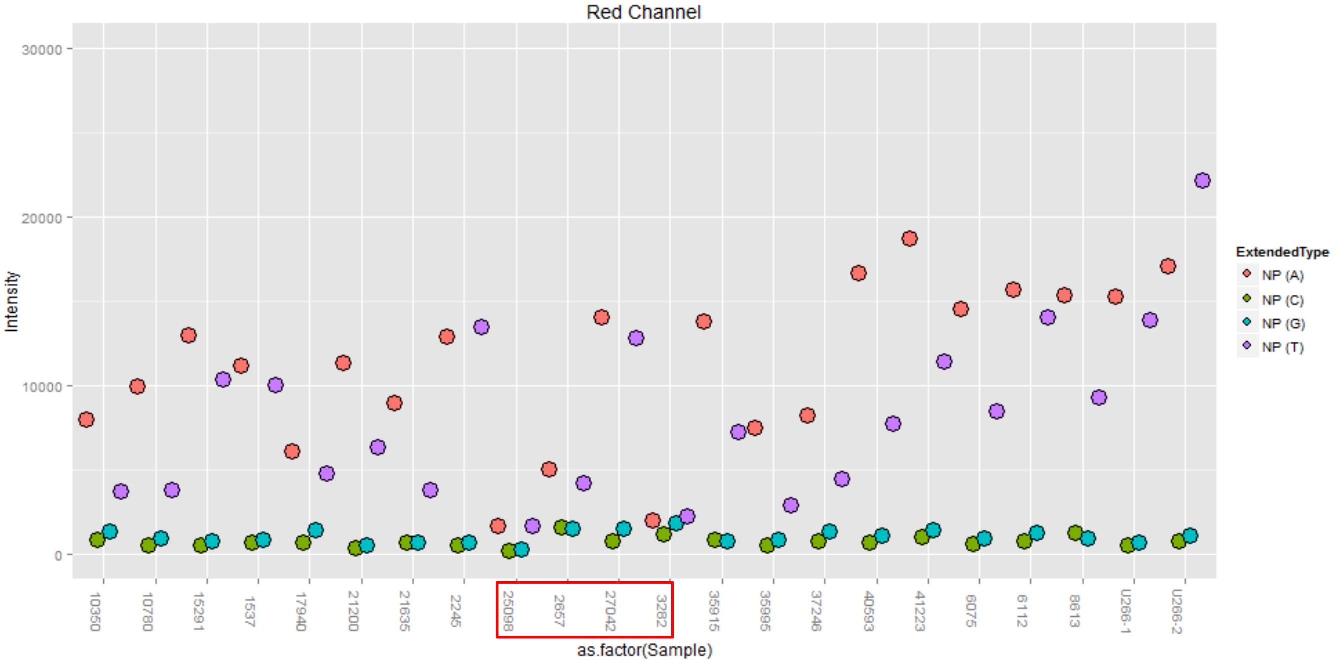

Supplement: Supplementary file 2 — 10.1186/s13104-015-1487-z Performance of HM450K sample-dependent control probes for failed and a subset of successful samples. Sample-dependent control probes measured background signal levels (negative; A), efficiency of bisulfite conversion using Infinium type I (B) and type II (C) probe designs, allele-specific extension of Infinium I and II probes (specificity; D) and restoration (E) and overall assay performance (non-polymorphic; F) on the red and green channels. Failed samples are boxed. [file 13104_2015_1487_MOESM2_ESM.pdf]
